# The impact of tizanidine, thiocolchicoside, and cyclobenzaprine on vascular function in ovariectomized rats

**DOI:** 10.55730/1300-0144.6115

**Published:** 2025-11-05

**Authors:** Sümeyye Nur GÜRSOY, Tolga ESMERLİGİL, Uğur Berkay İNC, Turhan DOST, Buket DEMİRCİ

**Affiliations:** Department of Medical Pharmacology, Faculty of Medicine, Aydın Adnan Menderes University, Aydın, Turkiye

**Keywords:** Endothelium, menopause, muscle relaxant, pain control, rational drug therapy

## Abstract

**Background/aim:**

Menopause is associated with increased vascular risk. This study investigated the effects of 3 centrally acting muscle relaxants—tizanidine (TZ), thiocolchicoside (TCC), and cyclobenzaprine (CBZ)—on vascular reactivity in ovariectomized (OVX) rats as a model of menopause.

**Materials and methods:**

Body weight, blood glucose, blood pressure, and heart rate were recorded, and the rats underwent oophorectomy surgery. Eight weeks after the operation, the rats were divided into 5 groups: sham operated rats, OVX rats, OVX rats treated with TZ (OVX + TZ), OVX rats treated with TCC (OVX + TCC), and OVX rats treated with CBZ (OVX + CBZ). All drug treatments were at a dosage of 2 mg/kg/day for 2 weeks. Isolated rat aortas were suspended in a tissue chamber. Vascular reactivity was assessed using increasing concentrations of phenylephrine, acetylcholine, and sodium nitroprusside, as well as potassium chloride at a concentration of 40 mM.

**Results:**

Body weight, phenylephrine sensitivity, and potassium chloride responses significantly increased with OVX. TZ and CBZ decreased body weight gain and ameliorated receptor-dependent contractile sensitivity. TZ and CBZ had calcium antagonistic effects on vascular smooth muscle. TZ deteriorated endothelial function.

**Conclusion:**

TZ cannot be considered a safe medication for patients with endothelial dysfunction. In high doses and for longer periods, TCC and CBZ might also have deleterious effects on vascular reactivity. These findings are noteworthy from the perspective of rational drug therapy.

## 1. Introduction

Menopause can span more than one third of a women’s life [1]. Estrogen has a cardioprotective role and is not produced in ovarian tissue after menopause, putting the vascular system at risk. Furthermore, the loss of estrogen initiates a rapid aging phase in menopausal women [2–5].

Muscle pain and discomfort are a common complaint among women as they transition through menopause [1,6]. Studies have shown that estrogen deficiency is associated with poorer outcomes for muscle mass, function, damage and repair, and energy metabolism in postmenopausal women [7]. Musculoskeletal disorders are the second leading disease class contributing to global disability after mental disorders in the aging women [6].

Tizanidine (TZ), thiocolchicoside (TCC), and cyclobenzaprine (CBZ) are different types of centrally acting muscle relaxants. They have been available without prescription for nearly half a century for pain control [8]. The drugs in this group have different mechanisms of action, side effects, dosages, and indications. TZ is a clonidine derivative and alpha-2 agonist. It is used to treat multiple sclerosis, brain and spinal cord injury, myofascial pain, and trigeminal neuralgia. It has been tested as an antiallodynic in a neuropathic pain rat model [9]. TCC is a semisynthetic derivative of colchicoside and a natural derivative of colchicine. It is used for the treatment of multiple sclerosis, cerebral palsy, and strokes that cause muscle spasm. It shows GABAergic activity and its antiinflammatory effects depend on inhibition of NF-κB-related pathways and COX-2 [10,11]. CBZ is structurally related to the tricyclic antidepressant amitriptyline. It is used to clinically decrease muscle spasm, hypercontractility, and acute musculoskeletal pain. It is known to mediate its effects centrally via inhibition of tonic somatic motor function, likely through modulation of noradrenergic and serotonergic systems [12]. TZ and CBZ have sedative properties [9,12], and they can be used to aid sleep.

Previous research has predominantly focused on the muscle relaxant properties of these drugs, but there is a significant knowledge gap regarding their impact on other organ functions. It is important to ensure that treatment agents are safe and do not adversely affect vascular function, especially in women that are already at increased risk for cardiovascular diseases. Therefore, we investigated the effects of these drugs on vascular function when used during menopause.

## 2. Materials and methods

### 2.1. Chemicals and drugs

TZ (Sirdalud tablet, Novartis, Istanbul, Türkiye), TCC (Muscoril ampoule, Bayer, Istanbul, Türkiye), CBZ (Flessi tablet, Menarini, Istanbul, Türkiye) were purchased from a local pharmacy. Phenylephrine hydrochloride (Phe), acetylcholine hydrochloride (Ach), sodium nitroprusside (SNP), potassium chloride (KCl), and chemical salts for Krebs–Henseleit solution were purchased from Sigma-Aldrich Chemicals (Interlab, İzmir, Türkiye).

### 2.2. Animals and experimental design

The study protocol was approved by the University of Aydin Adnan Menderes animal care and ethics committee (HADYEK 64583101/2023/02).

Female Wistar rats aged 8–12 weeks were obtained from the Experimental Animal Center of Aydin Adnan Menderes University. After shaving and cleaning, the rats were anesthetized with 50 mg/kg ketamine and 5 mg/kg xylazine. Ten rats were sham operated as normal controls (sham), while 40 rats underwent bilateral oophorectomy (OVX) [2,3]. The animals were left for 8 weeks for the development of menopause. OVX rats were then randomized into 4 subgroups: untreated (OVX), treated with TZ (OVX + TZ, 2mg/kg/day oral), treated with TCC (OVX + TCC, 2mg/kg/day intramuscular), and treated with CBZ (OVX + CBZ, 2mg/kg/day oral). The drugs were administered twice a day for 2 weeks. The total duration of the menopausal model was 10 weeks.

Rats were weighed weekly to adjust the muscle relaxant doses. Blood glucose was measured using a Clever Chek TD-4222 glucometer (TaiDoc Technology Corporation, Taiwan) without prior fasting because the aim was to assess glycemic status under normal metabolic conditions. Systolic blood pressure and heart rate were measured in conscious rats using the noninvasive tail cuff method (NIBP 200, Commat, Ankara, Türkiye).

### 2.3. Vascular reactivity studies

To reduce the number of animals used, 2 endothelium-intact thoracic aortic rings (approximately 3 mm in length) were obtained from each rat under ketamine (50 mg/kg) and xylazine (5 mg/kg) anesthesia immediately prior to euthanasia. A total of 6–8 rats per group were used for vascular reactivity experiments, yielding 12–16 aortic segments (2 rings per rat). The thoracic aorta was carefully excised, cleaned of surrounding connective tissue, and cut into rings. Each ring was immediately mounted in a 20 mL organ bath filled with Krebs–Henseleit solution (118.1 mM NaCl, 4.56 mM KCl, 1.22 mM CaCl_2_, 1.22 mM MgSO_4_, 1.1 mM KH_2_PO_4_, 25 mM NaHCO_3_, and 10.1 mM D-glucose), maintained at 37 °C and continuously aerated with a carbogen mixture (95% O_2_ and 5% CO_2_).

The rings were initially stretched to a resting tension of 2 g and allowed to equilibrate for 60 min. During this period, the bath solution was replaced every 15 min. Following equilibration, cumulative concentration–response curves to Phe (between 10^−9^ and 3 × 10^−4^ M) were generated. Each dose was added only after the response to the previous dose had reached a stable plateau. After the final dose of Phe, the tissues were washed twice with fresh Krebs–Henseleit solution and allowed to rest for 60 min, with the buffer being replaced every 15 min.

Subsequently, tissues were precontracted using 80% of the maximal Phe response. This value was calculated as: (maximum contraction to Phe − baseline tension) × 0.8 . The highest Phe dose was not used to avoid tissue exhaustion. Once a stable contraction was achieved, cumulative doses of Ach (between 10^−9^ and 3 × 10^−4^ M) were administered to assess endothelium-dependent relaxation. Doses were applied incrementally, and each was added after the response to the previous one had reached a plateau.

After Ach responses were recorded, tissues were washed twice and rested for another 60 min, again with solution changes every 15 min. The same protocol used for Ach was applied to assess endothelium-independent relaxation using SNP (between 10^−10^ and 3 × 10^−7^ M). For KCl induced contraction, a single concentration of 40 mM was added, and the response was recorded after reaching a plateau.

At the end of the experiment, the tissues were washed with Krebs–Henseleit solution and removed from the organ bath. The rings were placed on nonstick paper and left to dry for 24 h. The dry tissue weights were measured and used to calculate contractile responses as milligrams per dry tissue weight. Instead of using KCl-induced contractions for normalization, we used the dry weight of the aortic tissue because menopause-related changes can alter KCl responses and make them unreliable for normalization.

Isometric force was measured using a force transducer (MAY FDT 05, Commat Ltd. Ankara, Türkiye) and a data acquisition system (MP 150, Biopac Systems, Inc.) [2,3].

### 2.4. Statistical analysis

Body weight, blood glucose, tail blood pressure, and heart rate were evaluated for normality and Student’s t-test was used for pairwise comparisons (e.g., OVX vs. sham, OVX vs. each treatment group). Concentration–response curves were fitted by nonlinear regression with the simplex algorithm and the rings maximum responses (E_max_) and vascular sensitivity (pD_2_, −logEC50) values were calculated. E_max_ values of Phe and KCl were adjusted to the dry tissue weight of each ring. E_max_ and pD_2_ were evaluated for normality, and Student’s t-test was used for pairwise comparisons. Variance analysis (repeated measures ANOVA) was used to determine significance among the cumulative concentration curves. Data were expressed as mean ± standard error of mean (SEM), and p-values less than 0.05 were considered significant.

## 3. Results

### 3.1. Clinical findings

Development of menopause for 10 weeks and treatment of TZ, TCC, and CBZ for 2 weeks did not change blood glucose, tail blood pressure, and heart rate. Body weight increased in OVX (p < 0.001) and OVX + TCC groups (p < 0.001). TZ prevented weight gain (p < 0.01) and CBZ decreased body weight (p < 0.05). All the clinical finding are shown in Table 1.

### 3.2. Vascular reactivity results

The maximum response to Phe (E_max_) did not differ significantly among the groups. However, the sensitivity to Phe increased in the OVX group compared to the sham-operated group (p < 0.001). The sensitivity to Phe returned to normal in all 3 treatment groups. When the cumulative dose-concentration curves were evaluated, the TCC- and CBZ-treated groups remained significantly different (p < 0.05) from the sham-operated group.

The KCl response tended to increase in the OVX group compared to the sham-operated group (748.29 ± 43.15 vs. 628.10 ± 57.91, p < 0.05). The KCl response remained high in the TCC group (753.18 ± 61.05), while it decreased in the TZ (608.14 ± 41.62, p < 0.05) and CBZ (493.76 ± 40.53, p < 0.001) groups compared to the OVX group.

Ovariectomy did not deteriorate the Ach relaxation response (a marker of endothelial function). The pD_2_ value shifted slightly right, but not significantly. TZ treatment significantly deteriorated the Ach relaxation response (p < 0.05), and the cumulative dose–concentration curve was also significantly different from the sham group (p < 0.05). TCC and CBZ also decreased the Ach response, but not significantly.

SNP was used to assess smooth muscle function. OVX did not change the smooth muscle parameters during this time period. The E_max_ values of the groups were not significantly different. TZ decreased smooth muscle sensitivity (p < 0.05), and CBZ changed the cumulative dose–concentration curve significantly (p < 0.05).

**Figure 1:**
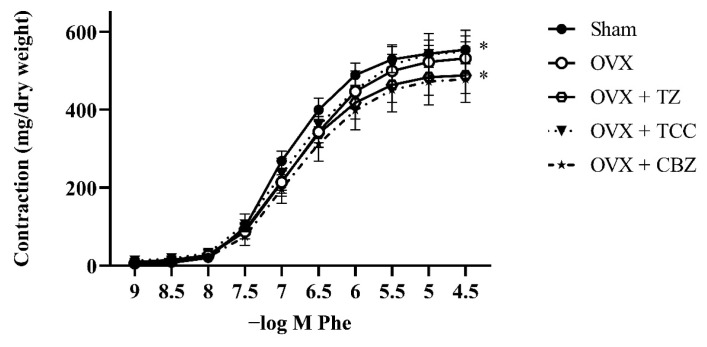
Concentration–response curves for Phe in endothelium-intact aortic rings of sham and of OVX rats. Some animals were treated with TZ (2 mg/kg/day), TCC (2 mg/kg/day), or CBZ (2 mg/kg/day) for 2 weeks. Values are presented as mean ± SEM from 14–16 vascular segments obtained from 7–8 rats. *Significance across the whole concentration–response curve (not at any single concentration), p < 0.05 for OVX + TCC vs sham group and OVX + CBZ vs sham group.

**Figure 2:**
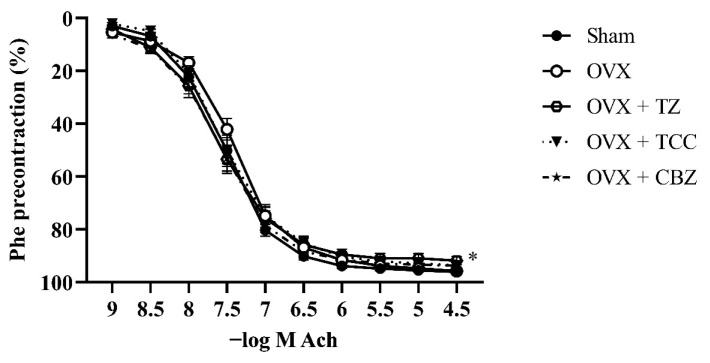
Concentration–response curves for Ach in intact endothelium aortic rings of sham and OVX rats. Some animals were treated with TZ (2 mg/kg/day), TCC (2 mg/kg/day), or CBZ (2 mg/kg/day) for 2 weeks. Values are presented as mean ± SEM from 14–16 vascular segments obtained from 7–8 rats. *Significance across the whole concentration–response curve (not at any single concentration), p < 0.05 for OVX + TZ vs sham group.

**Figure 3:**
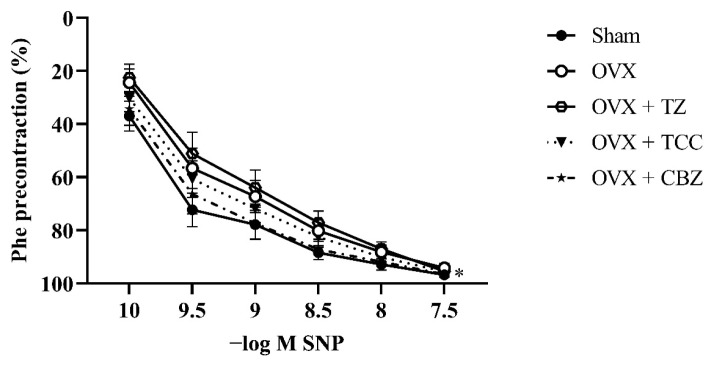
Concentration–response curves for SNP in intact endothelium aortic rings of sham and OVX rats. Some animals were treated with TZ (2 mg/kg/day), TCC (2 mg/kg/day), or CBZ (2 mg/kg/day) for 2 weeks. Values are presented as mean ± SEM from 12–16 vascular segments obtained from 6–8 rats. *Significance across the whole concentration–response curve (not at any single concentration), p < 0.05 for OVX + CBZ vs sham group.

The results of vascular reactivity are given in Table 2 and Figures 1, 2, and 3.

## 4. Discussion

The aim of this study was to investigate the safety of 3 centrally acting muscle relaxants, namely TZ, TCC, and CBZ, on the vascular system during menopause. Phe, an α_1_-adrenergic receptor agonist, was used to assess receptor-dependent contraction, whereas KCl, a depolarizing agent, was used to evaluate voltage-dependent contraction. The increased sensitivity to Phe (reflected by a higher pD_2_) and the trend toward increased KCl response in OVX rat aorta indicate that the vessels are more susceptible to contractile agents in this period. All the tested drugs decreased sensitivity to receptor-dependent contractility, as indicated by reduced pD_2_ values. Moreover, TCC and CBZ appear to reduce vascular sensitivity compared to the sham group. Additionally, TZ and CBZ also prevented the induction of voltage-dependent calcium channels by KCl (40 mM) on vascular smooth muscle, while TCC was ineffective.

We also investigated endothelium-dependent relaxant response to Ach and vascular smooth muscle relaxation with SNP. There are no well-controlled studies in the literature that document the safety and efficacy of TZ on the endothelium. Our study is the first to show that TZ has a detrimental effect on the endothelium as indicated by decreased E_max_ and decreased sensitivity of the vessels to SNP (Table 2). Therefore, TZ cannot be considered a safe medication for patients with endothelial dysfunction. Although not significant, TCC and CBZ also decreased the maximum relaxation of the vessel to Ach.

Menopause is a natural process that occurs in women when their ovaries stop producing estrogen and progesterone. It is linked to an elevated risk of cardiovascular and metabolic diseases, primarily resulting from the postmenopausal loss of estrogen [13,14]. This can lead to a number of health problems including widespread musculoskeletal pain. Unrelated to bone loss, musculoskeletal pain is a common complaint among postmenopausal women [1]. This leads to an intermittent self-medication with over-the-counter muscle relaxants, without a prescription.

Women lose the protective effect of estrogen on the cardiovascular system during menopause [2–4]. The vascular endothelium plays a crucial role in maintaining cardiovascular health by synthesizing and releasing vasodilator mediators such as nitric oxide (NO). There are studies showing that estrogen increases the bioavailability of NO, which is a vasodilator, antiagregant, and antiproliferative substance [13]. NO is produced by endothelial nitric oxide synthase (eNOS) activity in vascular endothelial cells. The expression of the gene encoding eNOS increases in endothelial cells exposed to estrogen [15]. Therefore, the vascular safety of medications is highly important in this context.

Menopause is associated with a significant increase in blood pressure and body mass index [4]. In our study, OVX resulted in increased body weight. TCC did not affect weight gain, but interestingly, 2 weeks of treatment with TZ prevented it and CBZ decreased weight gain. TZ and CBZ may be effective in preventing or reversing menopause-related weight gain. More research is needed to confirm these findings and to elucidate the underlying mechanisms.

Previous research has shown that rats develop high blood pressure 11 weeks after oophorectomy surgery [16]. Although we did not observe any significant elevation in blood pressure in our study using tail artery measurements, the increased sensitivity to Phe (reflected in a higher pD_2_) and the trend toward increased KCl response in OVX rat aorta indicate that the vessels are more susceptible to contractile agents in this period. Our findings are supported by a previous study that showed enhanced sensitivity to contractile agents in OVX rats [2].

TZ is derived from clonidine, but it has fewer side effects (e.g., hypotension and bradycardia) [17]. In our study, giving TZ to the rats did not produce any clinical side effects. Rodríguez-Palma et al. [9] reported that TZ (2 mg/kg/day) had a sex-dependent antiallodynic effect in neuropathy. They also suggest that activation of adrenergic receptors (α2B and α2A) and opioid receptors participate in the antiallodynic effect of TZ in female and male, respectively. The affinity of TZ for alpha-2 receptors in female explains our findings that TZ prevents the sensitivity of vascular smooth muscle to Phe. Similarly, TZ has been shown to be beneficial in reducing femoral artery vasospasm in rats [18]. TZ has been shown to exert its antinociceptive effects by decreasing the production of inflammatory cytokines, including TNF-α, IL-1β, and IL-6 [17].

In our study, TCC only normalized the increased sensitivity to Phe. It did not have any other effect on smooth muscle contraction or relaxation. A previous study found that 4 mg/kg of TCC was effective in preventing vasospasm following experimental subarachnoid hemorrhage in rabbits. However, this beneficial effect was attributed to the GABAergic activity of TCC [10].

CBZ is known to block alpha-1 adrenergic receptors, leading to vasodilation and a reflex increase in heart rate [12]. This mechanism may explain why we found that CBZ decreased the sensitivity of the aorta to Phe. Additionally, CBZ can block histamine H1 receptors and spinal cord 5-hydroxytryptamine (5-HT2) receptors [12,19]. Our results also suggest that CBZ has a calcium antagonistic effect on vascular smooth muscle through voltage-dependent channels.

The early closure of the ductus arteriosus and the development of pulmonary hypertension in a baby exposed to CBZ in utero have been interpreted as a potential effect of CBZ as a prostaglandin and NO inhibitor [20]. In a previous study, NO levels increased in macrophages incubated with CBZ, indicating its influence on NO regulation in different contexts [21]. CBZ also has anticholinergic properties as a tricyclic antidepressant [12]. The impairment effects of TCC and CBZ on endothelial function and vascular smooth muscle relaxation were not significant in our study at the dose and time course used. However, considering that muscle relaxants can be used in high doses and for longer periods to treat chronic spastic diseases, the deleterious effects of TCC and CBZ could be clinically important.

This study has limitations related to the use of young adult rats (8–12 weeks old) for oophorectomy. This does not fully replicate the age-related physiological context of human menopause. However, this model was intentionally chosen to isolate the effects of estrogen deficiency while minimizing the influence of age-associated vascular changes that could confound the interpretation of vascular responses.

In conclusion, this study provides important information regarding the potential vascular risks associated with the use of centrally acting muscle relaxants in postmenopausal women. TZ and CBZ have some calcium antagonistic effects on vascular smooth muscle. TZ may be beneficial for reducing menopausal weight gain and contractility, but its decrease in endothelial relaxant response is concerning. TCC and CBZ may also have deleterious effects on vascular reactivity. The effects of high doses and longer usage should also be considered when making rational drug therapy decisions.

## Figures and Tables

**Table 1:** Clinical features of all experimental groups. Values are mean ± SEM from 9–10 rats. OVX: ovariectomized, TZ: tizanidine (2 mg/kg/day), TCC: thiocolchicoside (2 mg/kg/day), and CBZ: cyclobenzaprine (2 mg/kg/day).

	Body weight (g)		Blood glucose (mmol/L)		Tail arterial blood pressure (mmHg)		Heart rate (beats/min)	
	Before	After	Before	After	Before	After	Before	After
**Sham**	236.31 ± 5.95	253.40 ± 6.96	9.20 ± 0.27	9.48 ± 0.34	132.02 ± 4.34	129.57 ± 3.96	351.64 ± 6.73	365.55 ± 9.20
**OVX**	234.80 ± 7.96	306.20 ± 10.94***	9.70 ± 0.19	8.81 ± 0.39	131.91 ± 2.78	125.75 ± 2.99	361.33 ± 15.62	364.57 ± 11.77
**OVX+TZ**	227.22 ± 6.23	269.54 ± 5.04##	9.48 ± 0.18	8.49 ± 0.41	128.68 ± 3.68	123.02 ± 4.17	370.53 ± 6.28	344.71 ± 12.03
**OVX+TCC**	244.33 ± 5.78	310.37 ± 8.45***	9.32 ± 0.28	9.80 ± 0.17	136.92 ± 2.19	135.6 ± 2.30	367.62 ± 11.15	375.03 ± 8.72
**OVX+CBZ**	223.46 ± 3.85	280.67 ± 9.26*	10.21 ± 0.45	9.08 ± 0.37	132.12 ± 3.81	133.26 ± 3.23	374.85 ± 8.88	372.76 ± 8.50

## p < 0.01 vs OVX;

* p < 0.05,

*** p < 0.001 vs. sham.

**Table 2:** Contractile responses to Phe and KCl (mg/dry tissue weight) and relaxant responses to Ach and SNP (percentage Phe precontraction) in endothelium-intact aortic rings of rats. E_max_ denotes maximum contraction and pD_2_ represents −logEC50. OVX: ovariectomized, TZ: tizanidine (2 mg/kg/day), TCC: thiocolchicoside (2 mg/kg/day), and CBZ: cyclobenzaprine (2 mg/kg/day). Values are presented as mean ± SEM from 12–16 vascular segments obtained from 6–8 rats.

	Phenylephrine		KCl	Acetylcholine		Sodium nitroprussid	
	Emax	pD2	Emax	Emax	pD2	Emax	pD2
**Sham**	553.84 ± 35.78	6.93 ± 0.03	628.10 ± 57.91	96.12 ± 0.63	7.61 ± 0.08	96.75 ± 1.05	10.25 ± 0.21
**OVX**	531.58 ± 42.23	7.75 ± 0.06***	748.29 ± 43.15	95.83 ± 0.77	7.42 ± 0.06	94.21 ± 1.70	9.73 ± 0.18
**OVX+TZ**	487.95 ± 46.65	6.85 ± 0.04###	608.14 ± 41.62#	91.84 ± 1.62*#	7.65 ± 0.09	95.43 ± 1.22	9.61 ± 0.20*
**OVX+TCC**	548.88 ± 54.98	6.78 ±0.06*###	753.18 ± 61.05	93.64 ± 1.20	7.54 ± 0.10	95.59 ± 0.83	9.95 ± 0.22
**OVX+CBZ**	478.28 ± 59.99	6.78 ± 0.05*###	493.76 ± 40.53###	93.78 ± 1.52	7.61 ± 0.07	96.63 ± 1.10	10.10 ± 0.23

* p < 0.05,

** p < 0.01,

*** p < 0.001 vs. sham;

# p < 0.05,

### p < 0.001,

## p < 0.01 vs. OVX group.
